# Multicentric lymphoma in buffaloes in the Amazon region, Brazil

**DOI:** 10.1186/s12917-016-0845-y

**Published:** 2016-10-20

**Authors:** Cairo H S De Oliveira, José D Barbosa, Karine A Damasceno, Geovanni D Cassali, Carlos MC Oliveira, Rômulo C Leite, Jenner K P Reis

**Affiliations:** 1 Laboratório de Retroviroses, Departamento de Medicina Veterinária Preventiva, Escola de Veterinária, Universidade Federal de Minas Gerais, Avenida Antônio Carlos 6627, Belo Horizonte, Minas Gerais Brazil; 2 Setor de Medicina Veterinária Preventiva, Escola de Veterinária e Zootecnia, Universidade Federal de Goiás, Goiânia, Goiás Brazil; 3 Hospital Veterinário de Grandes Animais, Instituto de Medicina Veterinária, Universidade Federal do Pará, Castanhal, Pará Brazil; 4 Laboratório de Patologia Comparada, Instituto de Ciências Biológicas, Universidade Federal de Minas Gerais, Belo Horizonte, Minas Gerais Brazil

**Keywords:** Lymphoma, Buffalo, BIV, BLV, BoHV-6, Brazil

## Abstract

**Background:**

The presence of lymphoma in buffaloes was first reported in India in the 1960s. The disease is similar to Enzootic Bovine Leucosis (EBL) caused by *Bovine leukemia virus* (BLV) in cattle; however, according to our results and those of other studies, the etiology of these lymphomas in buffalo do not appear to be associated with BLV. The objectives of this study are to describe four cases of the disease in buffaloes belonging to the same herd in the Amazon region of Brazil and to perform a clinical-anatomopathological, immunohistochemical, and etiological study of the lymphomas.

**Results:**

Over a period of ten years, four buffaloes were observed presenting progressive weight loss, swelling of peripheral lymph nodes, and nodules in the subcutaneous tissue. Upon necropsy, whitish-colored tumor masses were observed in the form of nodules in the subcutaneous tissue, along with miliary nodules on the serosal surfaces of abdominal and thoracic organs and tumors in lymph nodes and other organs. Neoplastic lymphocyte proliferation was observed through histopathology. An immunohistochemical study revealed that the neoplasias were formed by proliferation of predominantly B lymphocytes. The presence of BLV genome was not detected in the lymphomas when using the real-time PCR technique, nor was it detected through immunohistochemical staining using monoclonal antibodies against two viral proteins. *Bovine herpesvirus 6* was not detected in the tumors. However, *Bovine immunodeficiency virus* (BIV) was detected in samples of lymphoma and in the lymph nodes and kidneys of one of the animals.

**Conclusions:**

The occurrence of lymphoma in buffaloes is reported for the first time in Brazil and is characterized by B-cell multicentric lymphoma. The etiology of the disease does not appear to be associated with BLV; however, the detection of BIV in samples of lymphoma from one sick animal deserves further study, considering the oncogenic potential of this virus.

**Electronic supplementary material:**

The online version of this article (doi:10.1186/s12917-016-0845-y) contains supplementary material, which is available to authorized users.

## Background

In Brazil, the buffalo population is estimated at roughly 1.3 million heads, with 66 % of these animals raised in the northern region of the country, mainly in the state of Pará [1]. The main breeds raised in Brazil are Murrah, Mediterranean, Jafarabadi and Carabao, which are intended for beef production, mainly extensive, but are also used for milk production in a semi-intensive system.

The buffaloes are commonly raised in the same manner as cattle because their appearance is phenotypically similar to this species. Many owners and veterinarians employ the same management tools used in cattle when raising buffaloes and forget the particularities of each species. Similarly, researchers transfer studies performed in cattle to the buffalo species without first conducting more detailed investigations.

For 10 years, our research group has observed the occurrence of a lymphoproliferative disease in buffaloes in the Amazon region, state of Pará, Brazil. Lymphoma is a malignant neoplasm of the immune system characterized by neoplastic proliferation of lymphocytes, which causes the formation of tumor masses in various organs. In buffaloes, the occurrence of lymphoma has been reported since 1967 in India [2].

The lesions are characterized by floccules (miliary nodules), nodules, and papillae of continuous growth and velvety appearance on the parietal and visceral surfaces of the serosal membranes of organs. The involvement of lymph nodes occurs through mild tumefaction with varying degrees of swelling [3, 4].

The etiology of this illness in buffaloes is still unclear, and due to the multicentric character of the tumors, some authors suggest the involvement of a viral agent [5]. In cattle, the emergence of lymphomas are associated with infection by *Bovine leukemia virus* (BLV), which causes Enzootic Bovine Leucosis (EBL), in which one to five percent of infected animals develop lymphoma, 20 to 30 % develop persistent lymphocytosis, and the majority remain asymptomatic for long periods [6, 7].

More recent studies have described the occurrence of lymphoma in buffaloes in Venezuela [8] and India [9]. In these studies, the authors discussed the conditions as being cases of EBL but failed to demonstrate the presence of this virus in the sick animals.

Our group has detected the presence of some viruses infections in buffaloes in Brazil including *Bovine herpesvirus 6* (BoHV-6) but no causal link to any diseases has so far been established [10]. *Bovine immunodeficiency virus* (BIV) was studied in 607 samples of DNA taken from the whole blood of buffaloes in Brazil and was detected in 4.4 % of cases using molecular biology techniques [11]. In cattle, BIV has been associated with the occurrence of lymphoma and lymphadenopathy in experimentally inoculated animals [12], but in buffaloes their role is unknown.

A closer study of the etiology of lymphomas in buffalo has not been undertaken, and few reports of this disease can be found in the world literature. In Brazil, the occurrence of lymphoma in buffaloes has never been reported. The objectives of this study were to describe four cases of lymphoma in buffaloes from one herd in the Amazon region and to conduct a clinical-anatomopathological, immunohistochemical, and etiological study of this disease.

## Methods

### Animals

Four buffaloes manifesting clinical symptoms indicative of lymphoma (clinical cases 01, 02, 03 and 04), belonging to a property in the Amazon region (property A-PA), were studied. The property was located in the city of Castanhal, state of Pará, Brazil (1°18'12.4"S 47°56'30.8"W), and breeding of cattle and buffaloes was used for reproduction.

Epidemiological data, such as breed, age, sex and breeding system, were obtained through technical evaluations and informal consultations with handlers and the owner. The owner allowed the study and has signed a consent term.

All procedures and animal handling followed the ethical principles in animal experimentation, adopted by the Ethics Committee in Animal Experimentation of UFMG/CEUA, under Protocol n° 133/2012.

### Clinical and anatomopathological study

The animals were clinically evaluated for the presence and location of tumor nodules, enlargement of peripheral lymph nodes, appetite, and body condition.

The four animals were subjected to an anatomopathological exam in which fragments of tumor tissue were collected and fixed in 10 % formalin and sent to the Animal Pathology Department of the Animal Health Project in the Amazon of the Federal Rural University of Rio de Janeiro (Universidade Federal Rural do Rio de Janeiro), where they were processed routinely for histology and embedded in paraffin. The fragments were sectioned at 5 μm, stained with hematoxylin and eosin (HE) and evaluated through optical microscopy. In clinical case 04, fragments of the kidneys, lymph nodes, salivary gland, testis, liver, and spleen were also collected.

### Immunohistochemical study

An immunohistochemical examination was performed on the tumor and organ fragments of animal 04 to characterize the type of lymphocytes present in the lymphomas.

For the immunohistochemical study, 4-μm sections were placed on gelatinized slides, and the antigenic sites were recovered with the solution Dako Cytomation Target Retrieval Solution, Citrate pH 6.0 (Dako, Carpinteria, CA, USA), before overnight incubation with anti-CD79a antibodies (marker for B lymphocytes – clone HM47/A9, DBS, Pleasanton, CA, USA), at a dilution of 1:500, and anti-CD3 (marker for T lymphocytes – clone CD3-12, University of California, Davis, CA, USA), at a dilution of 1:200. Subsequently, the commercial secondary antibody Dako Advance HRP (Horseradish Peroxidase) Link (Dako, Carpinteria, CA, USA) was added, along with the enzyme Dako Advance HRP Enzyme, the reaction with DAB (3,3’-Diaminobenzidine, Dako Liquid DAB + Substrate Chromogen System) was revealed, and counter staining with Mayer’s Hematoxylin was performed.

To evaluate the presence of BLV viral proteins, immunohistochemical analysis was performed with monoclonal antibodies against the gp51 glycoproteins of the viral envelope (VMRD, Pullman, WA, USA), at a dilution of 1:100 and against the p24 protein of the viral capsid (VMRD, Pullman, WA, USA), at a dilution of 1:500. For the positive control of the reactions, FLK (fetal lamb kidney) cells continuously infected with BLV were grown on glass slides for 48 hours, fixed with acetone for 30 minutes, and processed together with the tumor fragments using a method similar to the one described above.

The slides were observed under an optical microscope for qualitative assessment of the presence or absence of staining by specific antibodies.

### Etiologic study

Three agents, BLV, BoHV-6 and BIV, were studied using PCR in fragments of lymphoma and organs from animal 04.

The tissue samples embedded in paraffin were sectioned at 5 μm and subjected to DNA extraction using a QIAamp DNA Mini Kit (Qiagen, Germany) according to the manufacturer’s recommendations for formalin-fixed, paraffin-embedded (FFPE) samples.

Frozen samples of tumor fragments from animal 04 were also subjected to DNA extraction with the above mentioned kit according to the manufacturer’s recommendations for fresh tissue samples.

The DNA samples were subjected to seminested PCR for BIV to detect the *pol* gene – the region that encodes the DNA polymerase enzyme – amplifying a 385-bp fragment in the outer reaction and a 154-bp fragment in the internal reaction, according to previously standardized methodology [13]. Real-time PCR was performed (qPCR) for BLV to detect the LTR gene, amplifying a 120-bp fragment according to a previous study [14]. Lastly, BoHV-6 was investigated by means of seminested PCR for the *pol* gene [10].

A positive sample for the BIV seminested PCR was cloned from the external reaction in pGEM®-T Easy Vector System II (Promega, EUA) according to the manufacturer’s recommendations, transformed and multiplied in DH5alpha bacteria. Two plasmid clones were extracted using a miniprep kit (Wizard Plus SV Minipreps DNA Purification Systems, Promega, USA). The region of the insertion (385 bp) in the plasmid was subjected to nucleotide sequencing using the seminested PCR external reaction primers in an ABI 3130 sequencing machine using Big Dye chemistry and POP7 polymer (Applied Biosystems, USA).

The sequence obtained (isolated Castanhal04) was evaluated for quality and edited for contig assembly on the web at http://www.biomol.unb.br/phph/. The sequence was blasted to evaluate the degree of similarity with the reference sequence for BIV in GenBank (GenBank database, National Center for Biotechnology Information), access code M32690.1 (isolated R-29), along with the isolates OK (GenBank: U34389.1), FL112 (GenBank: L06524.1) and FL491 (GenBank: L06525.1), which were aligned using the ClustalW method in the Mega5.2 program.

## Results

**Fig. 1:**
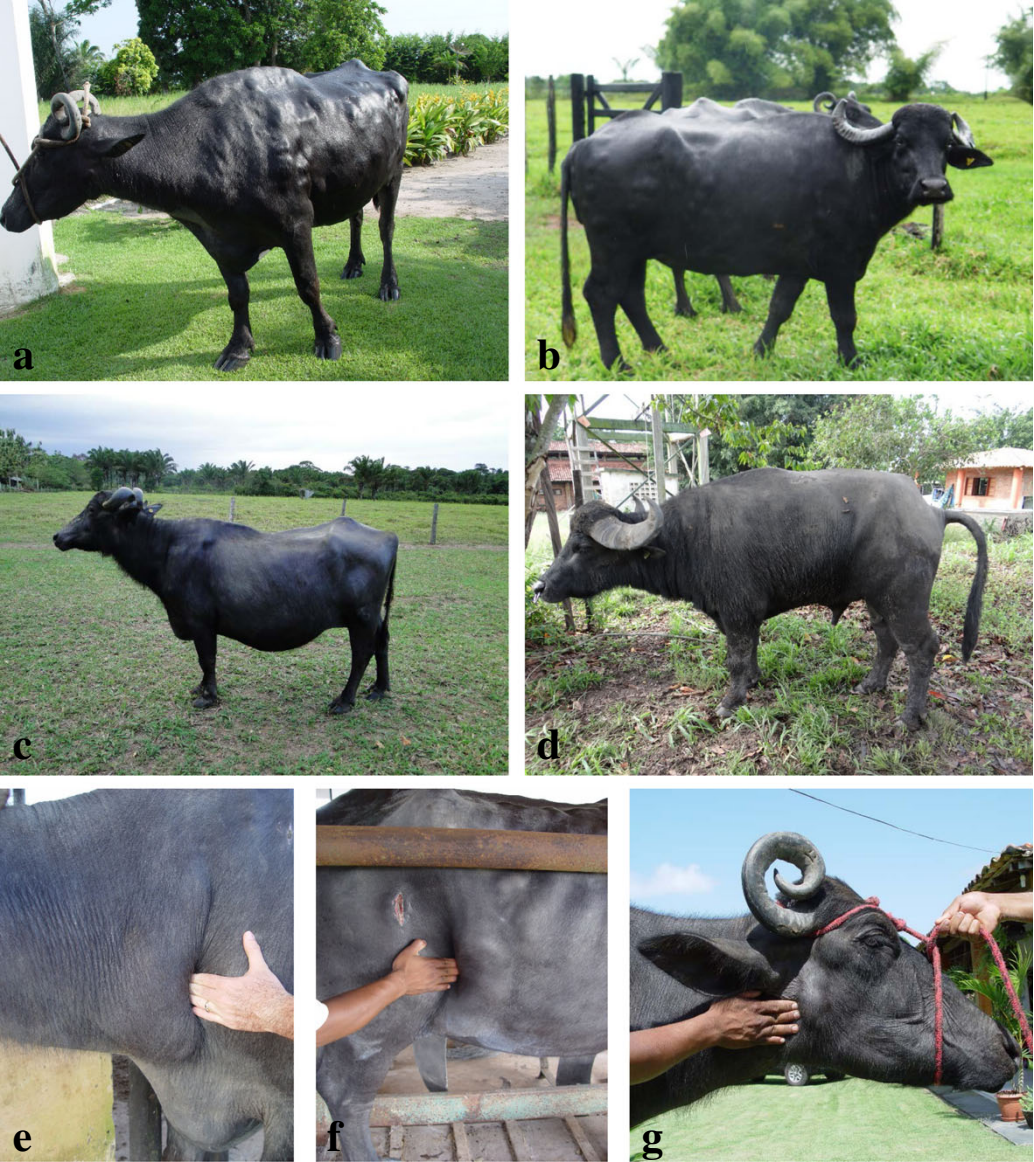
Lymphoma in buffaloes. **a** Animal 01 presenting subcutaneous nodules and weight loss. **b** Animal 02. **c** Animal 03 with swelling in the abdominal region. **d** Animal 04 with tenesmus and stranguria. Lymphadenopathy: **e** superficial cervical lymph node (pre-scapular) - animal 02, **f** subiliac lymph node (pre-crural) and **g** parotid lymph node - animal 01

In 2004, two buffaloes were observed presenting progressive weight loss, enlarged lymph nodes ranging from 2 to 20 times their normal size, and nodules of varying sizes in the subcutaneous tissue. The first case was observed in a female buffalo of the Murrah breed (01), and the second in an animal of the Mediterranean breed (02) (Fig. 1a and b).

In 2010, one Murrah female buffalo (03) was observed presenting the following main clinical signs: swelling of the abdominal region and progressive weight loss (Fig. 1c). In 2013, a male animal, of the Mediterranean breed (04), was observed presenting weight loss and great difficulty urinating and defecating (tenesmus and strangury), and upon rectal palpation, a tumor mass was noted at the entrance of the pelvic cavity on the right side (Fig. 1d).

All cases occurred on a single property where the buffaloes are raised together with cattle in a semi-intensive system. The farm maintains a buffalo herd of approximately 20 animals and a cattle herd of 30 animals, and over a 10-year period, four lymphoma cases in buffaloes were observed, including an animal that was introduced to this herd two years ago (animal 04). This history raised the hypothesis that the disease could have been transmitted horizontally. It was not possible to obtain information on the degree of kinship of the animals. Table 1 presents all evaluated cases and information on breed, sex, age and location of lymphoma lesions observed during necropsy.

**Table 1:** Epidemiological and pathological data of four clinical cases of lymphoma in buffaloes from one property (A-PA) located in the state of Pará, Amazon region, Brazil

Clinical cases	01	02	03	04
Sex	Female	Female	Female	Male
Breed	Murrah	Mediterranean	Murrah	Mediterranean
Age	Over 10 years	Over 10 years	Over 10 years	Over 10 years
Date of necropsy	11/07/2004	12/15/2004	11/12/2010	02/08/2013
Areas with the presence of neoplasia				
Subcutaneous	+++	+++		
Digestive system				
Serosa of the rumen			+++	++
Serosa of the omasum			+++	
Mucosa of the abomasum	+	+	+	
Serosa of the intestine			+++	
Liver	+	++	+++	
Gallbladder			+++	
Peritoneum		++	+++	
Omentum		+	+++	
Lymphatic system				
Parotid lymph node	+++	++		
Submandibular lymph node	+++			
Pre-scapular lymph node	+++			
Pre-crural lymph node	+++	++		
Iliofemoral lymph node	+++			++
Mediastinal lymph node	+	+		
Cardiorespiratory System				
Heart		+++		+++
Pericardial sac				++
Lung		+	+	
Parietal pleura			++	
Spleen	+		++	
Other findings			Presence of a large amount of yellowish fluid in the abdominal and thoracic cavity	Tumor masses located at the entrance of the pelvic cavity measuring 35 × 20 cm compressing the rectum and urethra, causing tenesmus and stranguria. Thickening of the bladder wall.

(+) Barely affected, (++) moderately affected and (+++) very affected

After identification of the disease, the animals quickly progressed to a state of great debility, with anorexia, cachexia, collapse and evolution of the nodules observed in the subcutaneous tissue and peripheral lymph nodes.

**Fig. 2:**
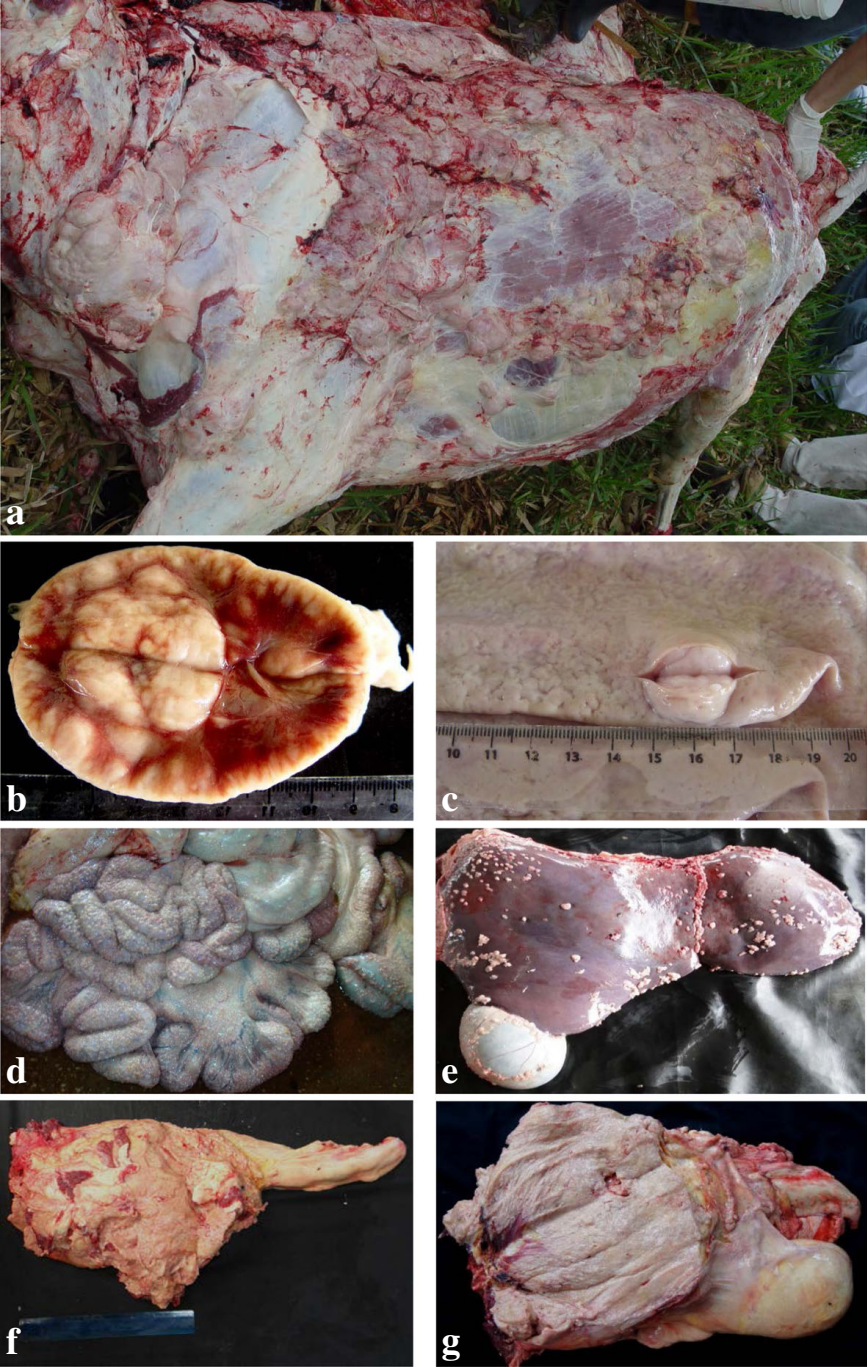
Macroscopic lesions in buffaloes with multicentric lymphoma. **a** Subcutaneous tissue with whitish nodules of different sizes and swelling of pre-scapular lymph node. **b** Surface of a lymph node with the presence of a tumor mass. **c** Tumor nodule in the abomasum. **d** Miliary nodules in the serosa of the intestines and mesentery. **e** Miliary nodules on the serosa of the liver and gallbladder. **f** Tumor mass measuring 35 x 20 cm located at the entrance of the pelvic cavity of a buffalo. **g** Surface of the tumor mass seen in **f**, characterized by a whitish coloration and fatty appearance. **a**-**c** from animal 01, **d**-**e** from animal 03 and **f**-**g** from animal 04

At the necropsy of animals 01 and 02, whitish nodules were observed, with sizes varying from 3 to 10 cm in diameter, diffuse along subcutaneous tissue (Fig. 2a). These nodules were soft in consistency when cut and were multilobular. The lymph nodes lost their normal morphology, and the surface appeared smooth and shiny with nodular areas of a whitish color (Fig. 2a and b).

In animal 03, the presence of tumor masses was observed in the serosa of the rumen, omasum, abomasum, intestines, liver, gallbladder, peritoneum, omentum, spleen, heart and pericardial sac. These masses appeared in the form of miliary nodules of continuous growth with a velvety or cauliflower appearance on the parietal and visceral surfaces of organs (Fig. 2d and e).

In animal 04, the presence of a large tumor mass was observed, measuring 35 × 20 cm, located at the entrance to the pelvic cavity (Fig. 2f and g). This mass was compressing the rectum and urethra, making defecation and urination difficult for the animal, and the mass infiltrated into the pelvic musculature in a small area. The consistency of the mass was friable, and the cut surface was multilobulated, with a whitish to beige color and the appearance of fat (Fig. 2g).

Lymphadenopathy was observed in all of the cases in peripheral (Fig. 1e, f and g) or internal lymph nodes. Tumor masses were observed in various organs, especially the abomasum (Fig. 2c), intestines and heart (Table 1).

**Fig. 3:**
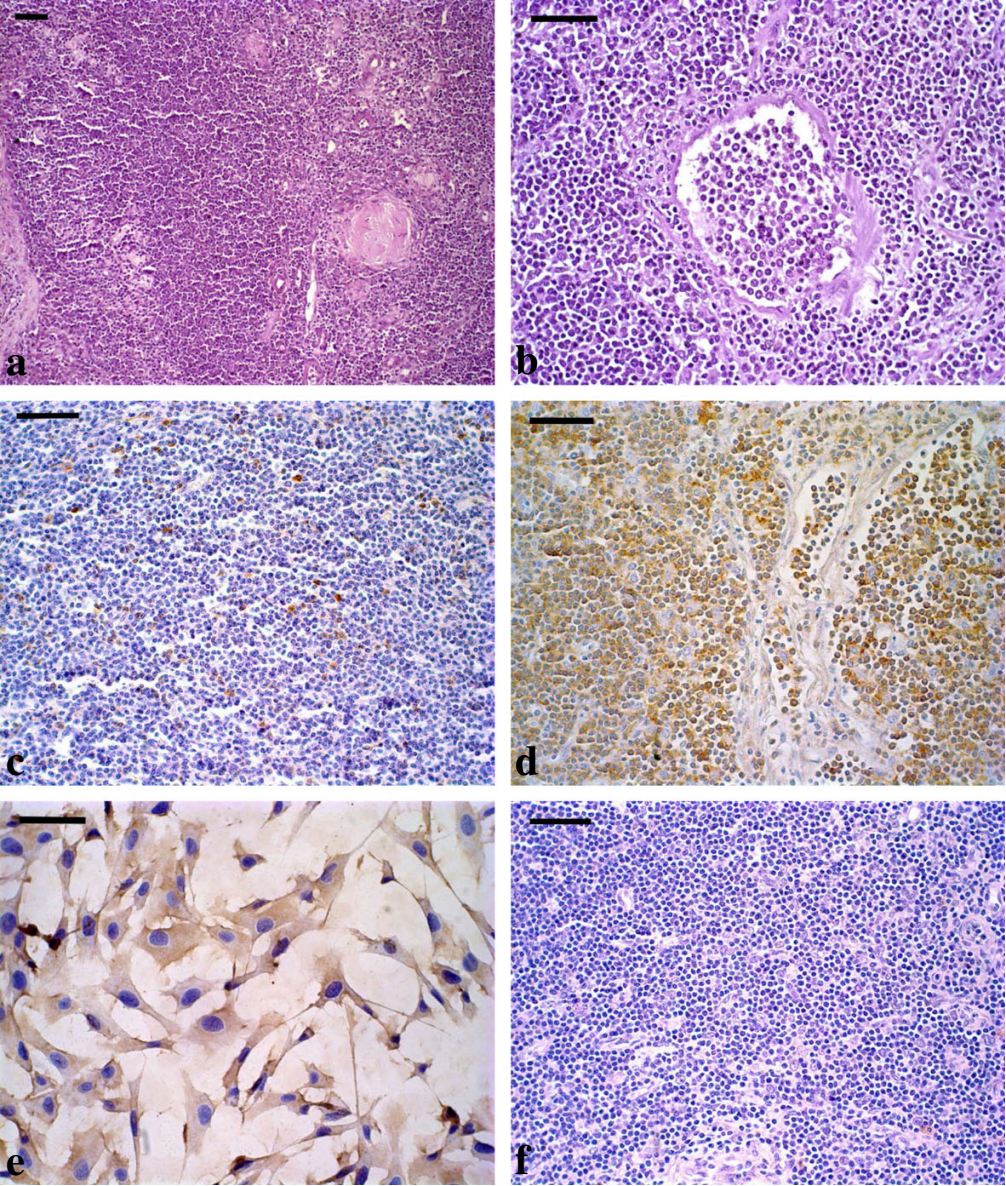
Histology and immunohistochemistry of clinical case 04. **a** Histological section of lymph node showing neoplastic lymphocytes, with loss of organ architecture. HE. **b** Tumor mass from pelvic cavity showing vascular invasion area for neoplastic cells. HE. **c** Membrane marking with anti-CD3 antibody. Immunohistochemical reaction with Mayer’s Hematoxylin counterstaining. **d** Labeling of membranes with anti-CD79 antibody. Immunohistochemical reaction with Mayer’s Hematoxylin counterstaining. **e** FLK cells with cytoplasmic staining using an anti-p24 antibody for BLV. Immunohistochemical reaction with Mayer’s Hematoxylin counterstaining. **f** Tumor mass from pelvic cavity with absence of staining using an anti-p24 antibody for BLV. Immunohistochemical reaction with Mayer’s Hematoxylin counterstaining. Bars = 50 μm

Histopathological analysis of the nodules and lymph nodes revealed the presence of tumor masses, characterized by the proliferation of round, discretely pleomorphic cells with little cytoplasm, large nuclei, and ovoid rounded basophilic shape, sometimes with loose chromatin and prominent nucleoli, compatible with lymphocytes. The presence of delicate fibrovascular tissue interweaving with the neoplastic process was observed. Eosinophilic areas were observed with cells in karyorrhexis and karyolysis, characterizing coagulative necrosis. The normal architecture of the lymph nodes was completely or partially replaced by neoplastic masses. Vascular invasion by neoplastic cells was also observed in the tumor masses (Fig. 3a and b).

When observed microscopically, whitish areas in the parenchyma of the liver and kidneys of animals 01 and 02 corresponded to areas of neoplastic tissue infiltration, which replaced the normal organ architecture.

An additional movie file shows the clinical signs, gross pathology and histopathology in more detail [see Additional file 1].



**Additional file 1:** Movie showing in details the clinical signs, gross pathology and histopathology of multicentric lymphoma in buffaloes from Brazil.


In the immunohistochemical analysis of pieces of tumor tissue from animal 04, the neoplastic cells showed immunoreactivity with the anti-CD79 antibody in the plasma membrane, characterizing the cells as B lymphocytes. Few cells expressed CD3, indicating the lack of T lymphocytes (Fig. 3c and d).

For BLV infection by immunohistochemistry analyses, FLK cells persistently infected with BLV were used. In these cells were observed production of viral p24 and gp51 proteins demonstrated by anti-p24 and anti-gp51 antibodies respectively (Fig. 3e). No staining was observed in neoplastic lymphocytes in lymph nodes and tumor fragments from animal 04 when the same antibodies were used (Fig. 3f). The DNA samples extracted from the frozen tumor fragments and the paraffin-embedded tissues of the liver, kidneys, salivary gland, lymph node, testis and tumor fragments tested negative for BLV through the qPCR technique and for BoHV-6 through seminested PCR. However, the samples of frozen and paraffin-embedded tumor fragments of the lymph nodes and kidneys tested positive for BIV through seminested PCR.

**Fig. 4:**
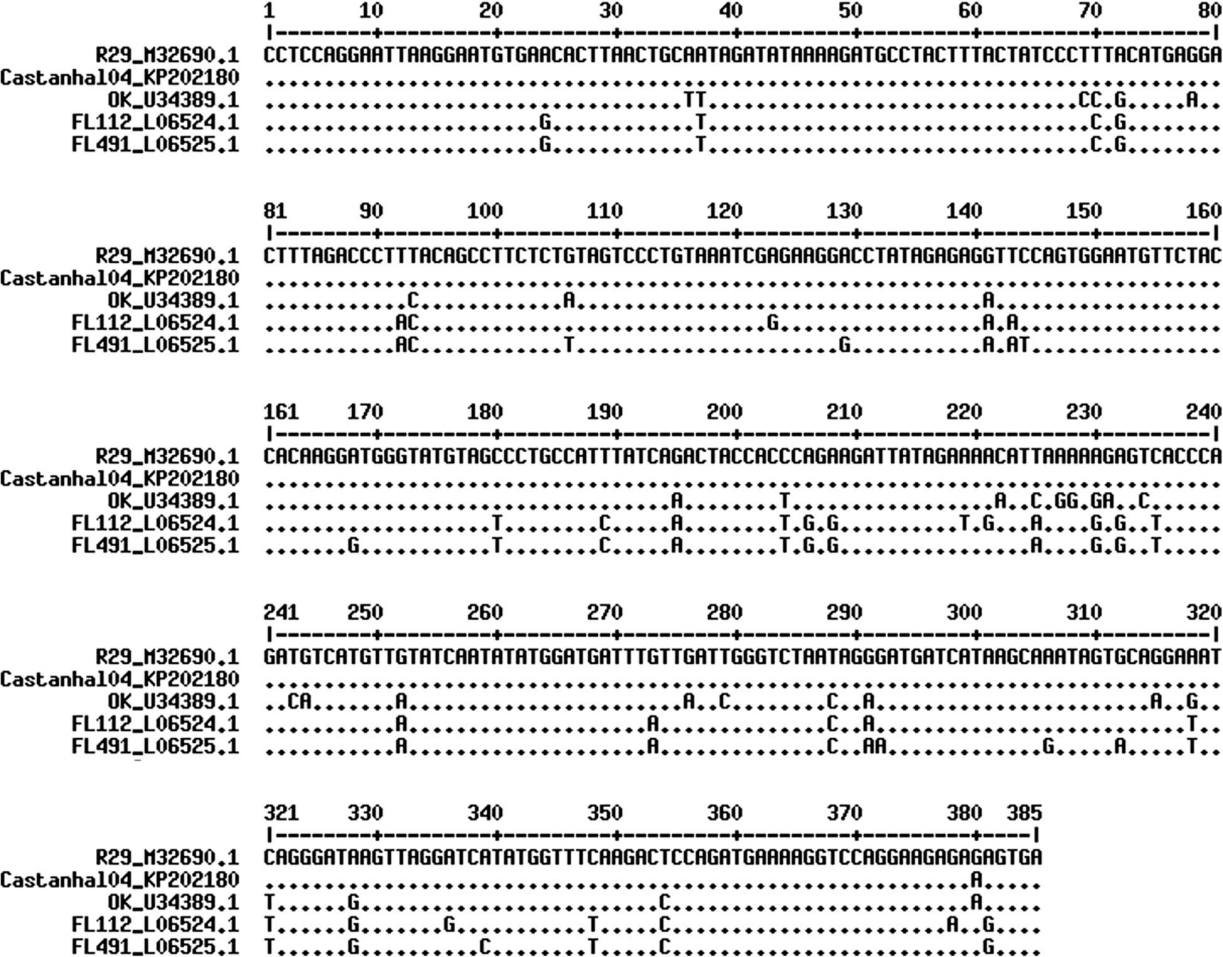
ClustalW alignment of the nucleotide sequence from part of the *pol* gene of BIV. Comparison of the isolated sequence of the sample of lymphoma of a buffalo from the state of Pará, Brazil, Castanhal04 isolate (GenBank: KP202180), with the reference sample for R-29 (GenBank: M32690.1), and the isolates for OK (GenBank: U34389.1), FL112 (GenBank: L06524.1) and FL491 (GenBank: L06525.1). Castanhal04 and R-29 showed 99 % similarity, with only one transition point mutation at position 380 of the alignment. The positions of the nucleotides are shown in accordance with the R-29 isolate (.), indicating the identity of the nucleotides

The nucleotide sequence obtained from the amplification of a 385-bp fragment of the *pol* gene of BIV from the frozen tumor sample of animal 04 (Castanhal04 isolate) was deposited in GenBank under access number KP202180. This sample showed 99 % similarity with the R-29 isolate of BIV in cellular culture, originally obtained from cattle with persistent lymphocytosis in 1972, 92 % with the OK isolate, 92 % with the FL112 isolate, and 91 % with the FL491 isolate. The alignment of nucleotides between the Castanhal04 isolate and the R-29 reference isolate showed only one transition point mutation at position 390, with the exchange of a G (guanine) for an A (adenine) (Fig. 4).

## Discussion

The occurrence of lymphoma has been reported in buffaloes only in India [2–5, 15, 16] and Venezuela [8]. This is the first report of the disease in buffaloes in Brazil.

The involvement of various organs and systems of the sick animals revealed the multicentric character of the disease, a fact also mentioned by other authors [8, 16]. In our study, only older animals were affected, suggesting that lymphoma in buffaloes is a chronic illness. In India, 17.8 % of adult animals were observed to have lymphoma lesions during slaughter; the rate was 1.23 % in animals up to two years old [3].

The pathological and histological lesions were similar to those observed by other authors [16]. Some authors reported the presence of large quantities of lymphoblasts and/or lymphocytes [5] and numerous mitotic figures in the histopathology of the tumor nodules. In this study, large quantities of lymphocytes and few cells in mitosis were observed. In the immunohistochemical analysis, the staining of most of the cells by the anti-CD79 antibody indicates that the tumors were characterized by the proliferation of B lymphocytes, a finding which has not yet been investigated in other studies.

In cattle with lymphoma caused by BLV, the macroscopic evaluation and histopathology of the tumors are similar to those observed in buffaloes in this study. In cattle, the tumors can occur in a multicentric form in various organs of the body, including the liver, lymph nodes, spleen, eyeballs, abomasum, rumen, and nervous system, among others. The histopathology is characterized by the proliferation of medium to large cells, lymphocytic and lymphoblastic in origin and with low mitotic indices. These cells have the phenotype of B lymphocytes, and necrotic areas are observed in the tumor masses [17, 18].

The etiology of this disease in buffaloes is still unclear. We suspect the involvement of a transmissible etiologic agent, as animal 04 presented the lymphoproliferative disease two years after its introduction into the herd of property A-PA, where the disease has been observed since the year 2004. Lymphoma in domestic animals can occur in spontaneous form associated with genetic and relatedness, although the degree of relationship of the sick buffaloes could not be accessed, the animals were obtained from different herds and does not seems to have any kinship. Additionally, lymphoma caused by genetic or undefined causes generally occur in younger animals, like sporadic leucosis in cattle not associated with BLV infection [19], unlike the cases in buffaloes that affected only older animals.

Due to the anatomopathological characteristics of the lymphomas in the buffaloes, some authors have diagnosed the disease as EBL [8, 9], but the involvement of BLV is unproven. Another important observation is that all of the animals in our study exhibited leukocytosis by lymphocytosis, like the other apparently healthy buffalo in the herd (data not shown), very similar to that observed in cattle infected with BLV [6, 7]. However, tumor and organ samples of a buffalo with lymphoma tested negative for BLV by qPCR and immunohistochemical analysis, which reinforces the hypothesis that this virus is not involved in the etiology of lymphoma in buffaloes. In a previous study, we used serological and molecular tests to evaluate 315 samples from buffaloes in Brazil, and the presence of BLV was not detected in any of the animals [20].

Gammaherpesviruses are known to have oncogenic potential [21, 22]. In humans, *Epstein-Barr virus* (EBV) is associated with the occurrence of Hodgkin lymphoma, Burkitt’s lymphoma, and diffuse lymphoma of large B cells, among others [23]. BoHV-6, a gammaherpesvirus initially detected in samples of bovine lymphomas caused by BLV [24], was not detected in samples of lymphoma from buffaloes evaluated in our study, despite its previous detection in the peripheral blood of other buffaloes from the same herd [10].

In contrast, samples of tumor fragments, kidneys and lymph nodes were positive for BIV, a retrovirus of the genus *Lentivirus*. This is the first detection of BIV in samples of lymphoma from buffaloes, and the sequence of nucleotides of Castanhal04 isolate had high similarity with the R-29 reference sequence. During a study of BIV pathogenesis, four calves, negative for BLV, were inoculated with the R-29 strain of BIV. One animal developed lymphadenopathy and monocytosis 160 days after inoculation. The calf died 203 days after inoculation, diagnosed with multicentric T cells lymphoma [12].

In cats, animals infected with the lentivirus *Feline immunodeficiency virus* (FIV) are five times more likely to develop B cell lymphoma [25]. The virus acts indirectly in the induction of tumors [26, 27], reducing the immune system’s surveillance mechanisms due to immunodeficiency, and may be involved in the polyclonal expansion of B cells, which predisposes the animal to mutations leading to oncogenesis [25]. Therefore, the importance of the detection of BIV in the buffalo species should be considered.

In slaughterhouses in the Amazon region, buffalo carcasses are being condemned as cases of tuberculosis or EBL due to the presence of neoplasia in various organs. Thus, the occurrence of lymphoma in buffalo seems to be larger than we actually realize.

## Conclusions

Buffaloes in the Amazon region have displayed a neoplastic disease characterized by multicentric B-cell lymphoma, but its pathogenesis is still unclear. The disease is observed in older animals, and the main clinical signs are progressive weight loss, lymphadenopathy, and the presence of nodules in the subcutaneous tissue. BIV was detected in the tumor samples, lymph nodes and kidneys of one sick animal. Thus the oncogenic role of this virus should be considered and investigated in future studies.

## Abbreviations

BIV: Bovine immunodeficiency virus
BLV: Bovine leukemia virus
BoHV-6: Bovine herpesvirus 6
bp: base pair
CEUA: Ethics Committee in Animal Experimentation
DNA: Deoxyribonucleic acid
EBL: Enzootic Bovine Leucosis
FFPE: Formalin-fixed, paraffin-embedded
FIV: Feline immunodeficiency virus
FLK: Fetal lamb kidney
HE: Hematoxylin and eosin
LTR: Long repeated terminal
PCR: Polymerase chain reaction
qPCR: Real time PCR
UFMG: Universidade Federal de Minas Gerais
μm: Micrometers

## References

[1] Produção da Pecuária Municipal (2013). Instituto Brasileiro de Geografia e Estatística. v.41.

[2] Bhattacharya P (1967). Lymphosarcoma in Indian buffaloes. Bull Off Int Epizoot..

[3] Singh CM (1968). Lymphosarcoma in Indian buffaloes. Bibl Haematol..

[4] Singh B, Singh KP, Parihar NS (1980). Lymphosarcomatous involvement of reproductive and endocrine organs in Indian buffalo. Zentralbl Veterinarmed A.

[5] Singh CM, Singh B, Parihar NS (1973). Pulmonary involvement in lymphosarcoma of Indian buffaloes. Bibl Haematol..

[6] Ghysdael J, Bruck C, Kettmann R (1984). Bovine leukemia virus. Curr Top Microbiol Immunol..

[7] Bartlett PC, Norby B, Byrem TM (2013). Bovine leukemia virus and cow longevity in Michigan dairy herds. J Dairy Sci..

[8] Vale-Echeto OE, Montiel-Urdaneta N, Simoes D (2009). Linfoma multicéntrico o linfosarcoma multicéntrico en Búfalo de agua (Bubalus bubalIs): Estudio Anatomopatológico. Reporte de un caso. Revista Científica.

[9] Chand N, Deshmukh S, Banga HS (2012). Bovine lymphosarcoma in a buffalo (Bubalus bubalis). Vet Pract..

[10] De Oliveira CH, de Oliveira FG, Gasparini MR (2015). Bovine herpesvirus 6 in buffaloes (Bubalus bulalis) from the Amazon region. Brazil. Trop Anim Health Prod..

[11] Albernaz TT, Leite RC, Reis JKP (2015). Molecular detection of bovine immunodeficiency virus in water buffaloes (Bubalus bubalis) from the Amazon retion. Brazil. Trop Anim Health Prod..

[12] Rovid AH, Carpenter S, Miller LD (1996). An atypical T-cell lymphosarcoma in a calf with bovine immunodeficiency-like virus infection. Vet Pathol..

[13] Rodrigues AS (2014). Detecção molecular do vírus da immunodeficiência bovina (BIV) em bovinos do estado de Minas Gerais. 59f. Dissertação (Mestrado em Ciência Animal) - Escola de Veterinária.

[14] Jimba M, Takeshima SN, Murakami H (2012). BLV-CoCoMo-qPCR: a useful tool for evaluating bovine leukemia virus infection status. BMC Vet Res..

[15] Gupta PP, Singh B, Gill BS (1977). Some uncommon neoplasms of Indian water buffaloes (Bubalus bubalis). Zentralbl Veterinarmed A..

[16] Singh B, Singh KP, Parihar NS (1979). Clinicopathological studies on lymphosarcoma in Indian buffaloes (Bubalus bubalis). Zentralbl Veterinarmed A..

[17] Chiba T, Hiraga M, Aida Y (1995). Immunohistologic studies on subpopulations of lymphocytes in cattle with enzootic bovine leukosis. Vet Pathol..

[18] Malatestinic A (2003). Bilateral exophthalmos in a Holstein cow with lymphosarcoma. Can Vet J..

[19] Grünberg W, Eisenberg SW (2013). Atypical form of sporadic bovine leukosis (SBL) in the Netherlands. Vet Rec..

[20] De Oliveira, C. H. S., Resende, C. F., Oliveira, C. M. C., Barbosa, J. D., Fonseca Junior, A. A., Leite, R. C., Reis, J. K. P. (2016). Absence of Bovine leukemia virus (BLV) infection in buffaloes from Amazon and southeast region in Brazil. Prev Vet Med. http://dx.doi.org/10.1016/j.prevetmed.2016.05.002 10.1016/j.prevetmed.2016.05.002 27317318

[21] Ackermann M (2006). Pathogenesis of gammaherpesvirus infections. Vet Microbiol..

[22] Cesarman E (2014). Gammaherpesviruses and lymphoproliferative disorders. Annu Rev Pathol..

[23] Thorley-Lawson DA, Hawkins JB, Tracy SI (2013). The pathogenesis of Epstein-Barr virus persistent infection. Curr Opin Virol..

[24] Rovnak J, Quackenbush SL, Reyes RA (1998). Detection of a novel bovine lymphotropic herpesvirus. J Virol..

[25] Beatty J (2014). Viral causes of feline lymphoma: retroviruses and beyond. Vet J..

[26] Beatty JA, Lawrence CE, Callanan JJ (1998). Feline immunodeficiency virus (FIV)-associated lymphoma: a potential role for immune dysfunction in tumourigenesis. Vet Immunol Immunopathol..

[27] Schmiedt CW, Grimes JA, Holzman G (2009). Incidence and risk factors for development of malignant neoplasia after feline renal transplantation and cyclosporine-based immunosuppression. Vet Comp Oncol..

